# Osmotic diuresis by SGLT2 inhibition stimulates vasopressin‐induced water reabsorption to maintain body fluid volume

**DOI:** 10.14814/phy2.14360

**Published:** 2020-01-28

**Authors:** Takahiro Masuda, Shigeaki Muto, Keiko Fukuda, Minami Watanabe, Ken Ohara, Hermann Koepsell, Volker Vallon, Daisuke Nagata

**Affiliations:** ^1^ Division of Nephrology Department of Internal Medicine Jichi Medical University Shimotsuke Tochigi Japan; ^2^ Department of Molecular Plant Physiology and Biophysics Julius‐von‐Sachs‐Institute of Biosciences University of Würzburg Würzburg Bavaria Germany; ^3^ Division of Nephrology and Hypertension Department of Medicine and Pharmacology University of California San Diego &VA San Diego Healthcare System San Diego CA USA

**Keywords:** bioimpedance analysis, glucosuria, SGLT2 inhibition, vasopressin, water reabsorption

## Abstract

Most of the filtered glucose is reabsorbed in the early proximal tubule by the sodium‐glucose cotransporter SGLT2. The glycosuric effect of the SGLT2 inhibitor ipragliflozin is linked to a diuretic and natriuretic effect that activates compensatory increases in fluid and food intake to stabilize body fluid volume (BFV). However, the compensatory mechanisms that are activated on the level of renal tubules remain unclear. Type 2 diabetic Goto‐Kakizaki (GK) rats were treated with vehicle or 0.01% (in diet) ipragliflozin with free access to fluid and food. After 8 weeks, GK rats were placed in metabolic cages for 24‐hr. Ipragliflozin decreased body weight, serum glucose and systolic blood pressure, and increased fluid and food intake, urinary glucose and Na^+^ excretion, urine volume, and renal osmolar clearance, as well as urine vasopressin and solute‐free water reabsorption (TcH2O). BFV, measured by bioimpedance spectroscopy, and fluid balance were similar among the two groups. Urine vasopressin in ipragliflozin‐treated rats was negatively and positively associated with fluid balance and TcH2O, respectively. Ipragliflozin increased the renal membrane protein expression of SGLT2, aquaporin (AQP) 2 phosphorylated at Ser269 and vasopressin V2 receptor. The expression of SGLT1, GLUT2, AQP1, and AQP2 was similar between the groups. In conclusion, the SGLT2 inhibitor ipragliflozin induced a sustained glucosuria, diuresis, and natriuresis, with compensatory increases in fluid intake and vasopressin‐induced TcH2O in proportion to the reduced fluid balance to maintain BFV. These results indicate that the osmotic diuresis induced by SGLT2 inhibition stimulates compensatory fluid intake and renal water reabsorption to maintain BFV.

## Graphical abstract

We show in this study that SGLT2 inhibitor ipragliflozin induced a sustained glucosuria, diuresis, and natriuresis, with compensatory increases in fluid intake and vasopressin‐induced aquaporin‐2 phosphorylation and solute‐free water reabsorption in proportion to the reduced fluid balance to maintain body fluid volume These results indicate that the osmotic diuresis induced by SGLT2 inhibition stimulates compensatory fluid intake and renal water reabsorption to maintain body fluid volume.

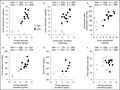

## 1 INTRODUCTION

Sodium‐glucose cotransporter 2 (SGLT2) inhibitors are oral antihyperglycemic drugs that suppress glucose reabsorption in the early proximal tubules (Vallon & Thomson, 2017). Recent large‐scale clinical trials have shown that SGLT2 inhibitors exhibit cardiorenal‐protective properties in type 2 diabetic patients (Neal et al., 2017; Perkovic et al., 2019; Wanner et al., 2016; Wiviott et al., 2019; Zinman et al., 2015). In particular, SGLT2 inhibitors induce robust benefits with regard to reducing heart failure hospitalization with and without atherosclerotic cardiovascular diseases or a prior history of heart failure (Zelniker et al., 2019), suggesting an important role of the diuretic property of SGLT2 inhibition. Because SGLT2 cotransports glucose with Na^+^ (in a 1:1 ratio) (Kanai, Lee, You, Brown, & Hediger, 1994; Vallon et al., 2011), SGLT2 inhibition decreases Na^+^ reabsorption in the early proximal tubule and the non‐reabsorbed glucose induces an osmotic diuretic effect (Lambers Heerspink, Zeeuw, Wie, Leslie, & List, 2013). In accordance, human and animals studies reported that SGLT2 inhibitors can modestly increase urinary Na^+^ excretion and urine volume (Ansary, Nakano, & Nishiyama, 2019; Masuda et al., 2017; Ohara et al., 2019).

We recently reported that SGLT2 inhibition with ipragliflozin induced a persistent natriuretic and diuretic tone, but euvolemia was maintained by an increase in fluid and food intake (Masuda et al., 2018). The only modest diuretic and natriuretic effects caused by SGLT2 inhibition induce homeostatic mechanisms that largely maintain body fluid status, which may help to attenuate the risk of body fluid depletion and acute kidney injury (AKI) (Nadkarni et al., 2017; Wiviott et al., 2019). In comparison, the more pronounced natriuretic and diuretic effects of loop diuretics decrease body fluid volume (Hu, Maslanik, Zerebeckyj, & Plato, 2012) and increase acute renal dysfunction in a dose‐dependent manner (Mullens et al., 2019).

Although adaptations in fluid and food intake constitute systemic effects to maintain volume homeostasis during SGLT2 inhibition, the compensatory mechanisms that are activated on the level of the renal tubules are less clear. The analysis of these tubular mechanisms would be facilitated by a rodent model in which SGLT2 inhibition induces a sustained diuretic effect. Some diabetic rodent models do not respond with a sustained increase in urine volume to chronic SGLT2 inhibition (Chen et al., 2016; Chung et al., 2019; Masuda et al., 2018). This is in part due to the fact that these models are more severely hyperglycemic and, as a consequence, basal glucosuria and urine flow rate are very high and do not significantly further increase in response to SGLT2 inhibitors. The latter is due to a strong reduction in blood glucose in response to SGLT2 inhibition in these models, which together with the associated lowering in GFR, reduces filtered glucose load to a similar extent as the drugs inhibit proximal tubular glucose reabsorption, such that glucosuria and urine flow rate remain largely unchanged, consistent with mathematical modeling (Layton, Vallon, & Edwards, 2016). In comparison, non‐obesity type 2 diabetic Goto‐Kakizaki (GK) rats only have mild hyperglycemia and in this regard mimic patients with type 2 diabetes and likewise show a sustained increase in glucosuria and urine volume in response to SGLT2 inhibition (Iuchi et al., 2017). We therefore examined the effects of the SGLT2 inhibitor ipragliflozin on renal water handling in diabetic GK rats.

## 2 MATERIALS AND METHODS

### 2.1 Experimental animals

The protocol of this study was approved by the Jichi Medical University Animal Ethics Committee. Non‐obesity type 2 diabetic GK rats at 4 weeks of age were purchased from the CLEA Japan Inc. GK rats were housed in a 12 hr:12 hr light:dark cycles in normal cages with free access to fluid and food (0.35% Na^+^, 1.01% K^+^, 4.92% fat, CLEA Rodent Diet CE2, CLEA Japan Inc.). GK rats at 18–22 weeks of age were randomly divided to receive normal food CE2 (Veh) or CE2 containing 0.01% ipragliflozin (Ipra) (Astellas Pharma Inc.) (Tahara et al., 2012a, 2012b) with free access to fluid and food, as described previously (Masuda et al., 2012, 2018). After 8 weeks of treatment, GK rats were put in metabolic cages to collect 24‐hr urine. Blood pressure was measured by a tail‐cuff method (Softron BP‐98A, Softron), as described previously (Masuda et al., 2012, 2018). This was followed by bioimpedance spectroscopy (BIS) using the ImpediVet BIS1 system (ImpediMed) to measure body fluid volume (total body water, extracellular fluid and intracellular fluid) at 8 weeks of treatment, as previously described (Chapman, Hu, Plato, & Kohan, 2010; Masuda et al., 2014, 2018). The BIS measurement was carried out during 2.5% isoflurane anesthesia (Dainippon Sumitomo Pharma Co. Ltd). Results were expressed as absolute values. Finally, blood samples were collected by cardiac puncture and the kidneys were taken under terminal anesthesia with isoflurane.

### 2.2 Plasma and urine analysis

The measurement of serum and urine parameters, including electrolytes, glucose, and osmolality, as well as urinary vasopressin concentrations was entrusted by the SRL laboratory (Masuda et al., 2018; Yasuoka et al., 2013). Urinary vasopressin concentration is a more reliable integrated index of vasopressin secretion than the concentration of vasopressin in a single blood sample (Matsui, Share, Wang, Crofton, & Brooks, 1983). Creatinine clearance (CCr) was calculated by the following formula: CCr (L/day) = urine creatinine concentration (mg/dl) × urine volume (L/24‐hr)/serum creatinine (mg/dl). Osmolar clearance, solute‐free water reabsorption, and electrolyte‐free water clearance were calculated by the following formulas: osmolar clearance = Uosm × UV/Sosm; solute‐free water reabsorption = osmolar clearance − UV; electrolyte‐free water clearance = UV × [1 − (UNa + Uk)/SNa] (UV: urine volume, Uosm: urine osmolality, Sosm: serum osmolality, UNa: urine Na, Uk: urine K, SNa: serum Na) (Dolgor et al., 1998; Huang, Pfaff, Serradeil‐Le Gal, & Vallon, 2000; Nguyen & Kurtz, 2005; Sansoe, Aragno, Smedile, Rizzetto, & Rosina, 2009).

### 2.3 Western blot analysis

The membrane fraction obtained from whole kidneys was used for western blot analysis as described previously (Masuda et al., 2018; Sabolic et al., 2012; Vallon et al., 2011). Lysates at 40 µg/lane of proteins were resolved on Nu‐PAGE 4%–12% Bis–tris gels in MOPS buffer. Gel proteins were transferred to a polyvinylidene difluoride membrane (Hybond‐P, GE Healthcare) and immunoblotted with the primary antibodies: polyclonal rat SGLT2 (dilution 1:1,000) (Masuda et al., 2018; Sabolic et al., 2012), polyclonal rat SGLT1 (dilution 1:2000) (Balen et al., 2008; Masuda et al., 2018), glucose transporter 2 (GLUT2, dilution 1:5,000, ab95256, Abcam) (Masuda et al., 2018), aquaporin 1 (AQP1, dilution 1:1,000, AB2219, Millipore) (Montiel et al., 2014), aquaporin 2 (AQP2, dilution 1:200, AQP‐002, Alomone Lab) (Abdeen, Sonoda, El‐Shawarby, Takahashi, & Ikeda, 2014), phosphorylated (Ser269) AQP2 (dilution 1:1,000, p112‐269, PhosphoSolutions) (Yui, Sasaki, & Uchida, 2017), vasopressin V2 receptor (AVPR2, dilution 1:200, AVR‐012, Alomone Lab) (Chung et al., 2019), and mouse anti‐β‐actin (1:2,000, sc‐47778, Santa Cruz Biotechnology) (Maric‐Bilkan, Flynn, & Chade, 2012), and followed by treatment with horseradish peroxidase‐conjugated secondary antibody. Protein expression was detected autoradiographically by ECL Plus (Amersham Pharmacia). Densitometric analysis was performed using the ImageJ Software (version 1.52a, National Institutes of Health).

### 2.4 Statistical analysis

Data are expressed as means ± *SE*. Statistical differences were analyzed by unpaired *t* tests to compare the two groups. *p* values less than .05 were considered to be statistically significant.

## 3 RESULTS

### 3.1 The SGLT2 inhibitor ipragliflozin increased food intake, fluid intake, and urinary fluid and Na^+^ excretion in diabetic GK rats

**Figure 1:**
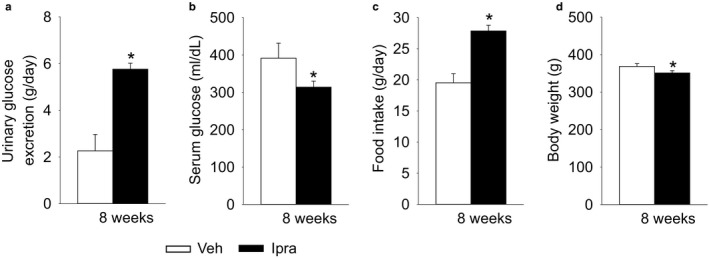
Ipragliflozin increased urinary glucose excretion in diabetic GK rats, associated with lower serum glucose and higher food intake. Effect of ipragliflozin on urinary glucose excretion (a), serum glucose (b), food intake (c), and body weight (d). Veh: normal diet (CE2, CLEA JAPAN Inc.); Ipra: normal diet containing ipragliflozin [100 mg/kg diet]. *p < .05 versus Veh. Values are expressed as means ± standard error; *n* = 8–11/group

Basal blood glucose and body weight (BW) before treatment were similar in the two groups of GK rats [blood glucose: Veh 194 ± 31 mg/dl vs. Ipra 191 ± 20 mg/dl, not significant (NS), BW: Veh 366 ± 6 g vs. Ipra 365 ± 7 g, NS]. At 8 weeks of treatment, ipragliflozin treatment led to higher absolute (Figure 1a) and fractional (Table 1) urinary glucose excretion versus vehicle and lower serum glucose levels (Figure 1b). This was associated with higher food intake (Figure 1c), such that body weight only declined slightly (Figure 1d) despite increased urinary glucose and thus calorie loss in response to ipragliflozin.

**Table 1:** Urine and blood parameters after 8‐week treatment of ipragliflozin

	Vehicle	Ipragliflozin
Kidney weight (g)	1.64 ± 0.05	1.77 ± 0.07
Urinary protein (mg/day)	56.5 ± 16.2	46.7 ± 8.9
Calculated urine osmolality (mOsmol/kgH_2_O)^a^	1529 ± 146	1,259 ± 56*
Urinary glucose (mEq/L)	411 ± 95	589 ± 28*
Urinary Na^+^ (mEq/L)	87 ± 17	48 ± 2*
Urinary K^+^ (mEq/L)	170 ± 27	89 ± 6*
Urinary Cl^−^ (mEq/L)	103 ± 18	55 ± 2*
Urinary Cl^−^ (mEq/day)	2.3 ± 0.1	3.0 ± 0.1*
Urinary urea nitrogen (mEq/L)	682 ± 98	398 ± 17*
Urinary urea nitrogen (mg/day)	421 ± 32	604 ± 21*
Sodium balance (mEq/day)	1.9 ± 0.6	1.9 ± 0.4
Hematocrit (%)	45.3 ± 0.8	47.1 ± 0.4*
Blood urea nitrogen (mg/dl)	19.5 ± 1.4	21.4 ± 0.8
Serum creatinine (mg/dl)	0.25 ± 0.01	0.27 ± 0.01*
Creatinine clearance (L/day)	3.8 ± 0.3	4.1 ± 0.2
Serum Na^+^ (mEq/L)	142 ± 1	144 ± 1*
Serum Cl^−^ (mEq/L)	101 ± 2	105 ± 1*
Serum K^+^ (mEq/L)	5.5 ± 0.4	5.1 ± 0.1
Fractional excretion of glucose (%)	16 ± 4	46 ± 3*
Fractional excretion of Na^+^ (%)	0.35 ± 0.03	0.44 ± 0.02*
Fractional excretion of fluid (%)	0.8 ± 0.1	1.3 ± 0.1*

a Calculated formula: 2*[urinary Na^+^ (mEq/L) + urinary K^+^ (mEq/L)] + urinary urea nitrogen (mEq/L) + urinary glucose (mEq/L).

* *p* < .05 versus Vehicle. Values are means ± *SE*; *n* = 4–11/group.

**Figure 2:**
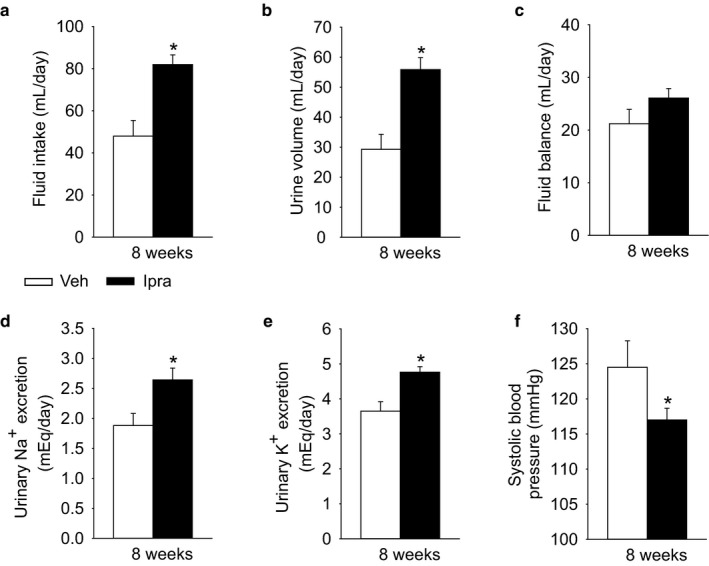
Ipragliflozin increased urinary fluid and Na^+^ excretion in diabetic GK rats, but did not significantly change fluid balance (fluid intake ‐ urine volume) due to an increase in fluid intake. Effect of ipragliflozin on fluid intake (a), urine volume (b), fluid balance (c), urinary Na^+^ excretion (d), and urine K^+^ excretion (e). At 8 weeks, ipragliflozin decreased systolic blood pressure (f). Veh: normal diet (CE2, CLEA JAPAN Inc.); Ipra: normal diet containing ipragliflozin [100 mg/kg diet]. *p < .05 versus Veh. Values are expressed as means ± standard error; *n* = 8–11/group

Ipragliflozin increased both fluid intake and urine volume to a similar extent (Figure 2a and b), such that fluid balance (fluid intake – urine volume) was also similar between the two groups (Figure 2c). Urinary excretions of Na^+^, Cl^−^, K^+^, and urea nitrogen were significantly increased by ipragliflozin treatment for 8 weeks (Figure 2d and e, Table 1), as expected based on the higher food intake. This was related to greater fractional excretion of Na^+^ and fluid, respectively (Table 1). Blood urea nitrogen and creatinine clearance were similar between the groups, arguing against major differences in GFR. The hematocrit and the concentrations of the main cation and anion in serum, Na^+^ and Cl^−^, were slightly but significantly higher in response to ipragliflozin (Table 1), potentially indicating a small reduction in circulating volume. This was associated with decreased systolic blood pressure during ipragliflozin treatment (Figure 2f), while sodium balance (sodium intake ‐ sodium excretion) (Table 1) and heart rate (Veh: 389 ± 27 min^−1^, Ipra: 345 ± 20 min^−1^, NS) were not significantly different between the groups.

### 3.2 Ipragliflozin increased urine vasopressin excretion in diabetic GK rats, which was positively associated with solute‐free water reabsorption

**Figure 3:**
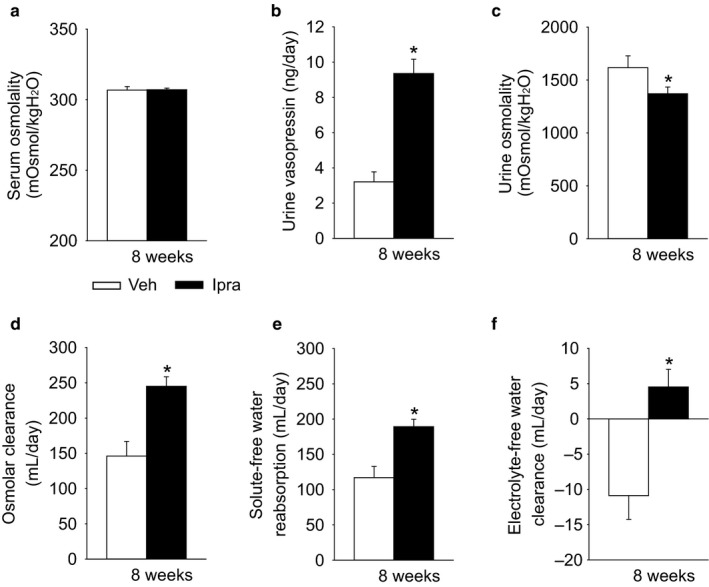
Ipragliflozin increased urine vasopressin and solute‐free water reabsorption in diabetic GK rats. Effect of ipragliflozin on serum osmolality (a), urine vasopressin (b), urine osmolality (c), osmolar clearance (d), solute‐free water reabsorption (e), and electrolyte‐free water clearance (f). Veh: normal diet (CE2, CLEA JAPAN Inc.); Ipra: normal diet containing ipragliflozin [100 mg/kg diet]. *p < .05 versus Veh. Values are expressed as means ± standard error; *n* = 8–11/group

Ipragliflozin did not change total serum osmolality, but the increase in serum Na^+^ and Cl^−^ concentrations (see above) was associated with increased urine vasopressin excretion (Figure 3a and b), while urine osmolality was lower in diabetic GK rats treated with ipragliflozin (Figure 3c). Among the determinants of urinary osmolality, the concentration of urinary Na^+^, urinary K^+^, and urinary urea nitrogen was significantly decreased by ipragliflozin, whereas the concentration of urinary glucose was significantly increased (Table 1). As a result, the urinary osmolality calculated by the formula (2*[urinary Na^+^ + urinary K^+^] + urinary urea nitrogen + urinary glucose) is significantly decreased by ipragliflozin (Table 1). Similarly, the direct measurement of urine osmolality is also significantly decreased by ipragliflozin (Veh 1617 ± 112 vs. Ipra 1,371 ± 64 mOsmol/kgH_2_O, *p* = .028) (Figure 3c). Ipragliflozin increased osmolar clearance, solute‐free water reabsorption, and electrolyte‐free water clearance (Figure 3d–f).

**Figure 4:**
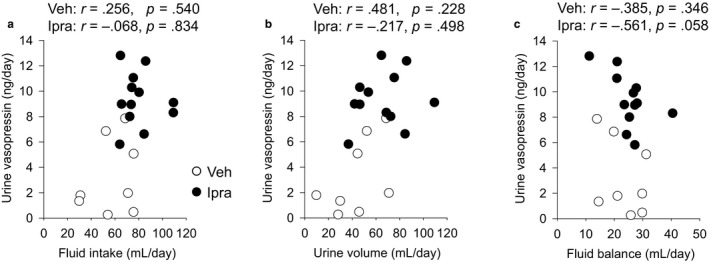
The relationship between fluid intake, urine volume, fluid balance, and urine vasopressin. Fluid intake and urine volume were not correlated with urine vasopressin (a and b). Fluid balance was negatively correlated with urine vasopressin with a stronger relationship in diabetic GK rats treated with ipragliflozin (c). Veh: normal diet (CE2, CLEA JAPAN Inc.); Ipra: normal diet containing ipragliflozin [100 mg/kg diet]. Values are expressed as means ± standard error; *n* = 8–12/group

**Figure 5:**
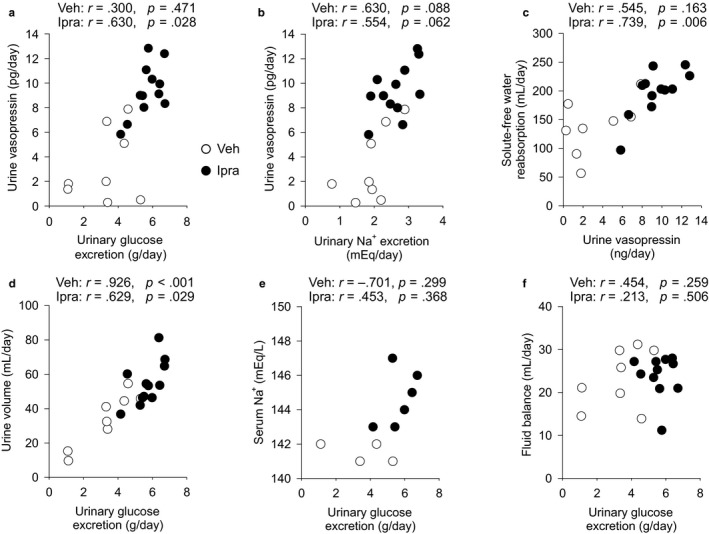
The relationship between urinary glucose and Na^+^ excretion and urine vasopressin. Urinary glucose excretion was positively correlated with urine vasopressin (a). Urinary Na^+^ excretion tended to be correlated with urinary vasopressin (b). Urine vasopressin in ipragliflozin‐treated diabetic GK rats was significantly and positively correlated with solute‐free water reabsorption (c). In ipragliflozin‐treated diabetic GK rats, urinary glucose excretion was positively and significantly correlated with urine volume (d), and there was a positive trend relationship between urinary glucose excretion and serum Na^+^ (e). Urinary glucose excretion in ipragliflozin‐treated rats was not associated with fluid balance (f). Veh: normal diet (CE2, CLEA JAPAN Inc.); Ipra: normal diet containing ipragliflozin [100 mg/kg diet]. Values are expressed means ± standard error; *n* = 4–12/group

To gain further functional insights, specific parameters were correlated with urine vasopressin excretion. Fluid intake (Veh: *r* = .256, NS; Ipra: *r* = −.068, NS) and urine volume (Veh: *r* = .481, NS; Ipra: *r* = −.217, NS) were not correlated with urine vasopressin (Figure 4a and b). Fluid balance tended to be negatively correlated with urine vasopressin with a stronger relationship in ipragliflozin‐treated diabetic rats (Veh: *r* = −.385, NS; Ipra: *r* = −.561, *p* = .058) (Figure 4c). These results are consistent with the notion that the lower fluid balance in response to ipragliflozin increased vasopressin secretion in an effort to stabilize body water. Urinary glucose excretion in ipragliflozin‐treated diabetic rats was positively and significantly correlated with urine vasopressin (Veh: *r* = .300, NS; Ipra: *r* = .630, *p* = .028) (Figure 5a). Urinary Na^+^ excretion (Veh: *r* = .630, *p* = .088; Ipra: *r* = .554, *p* = .062) tended to be related to urinary vasopressin (Figure 5b). Urine vasopressin in ipragliflozin‐treated diabetic rats was significantly and positively related to solute‐free water reabsorption (Veh: *r* = .545, NS; Ipra: *r* = .739, *p* = .006) (Figure 5c). In ipragliflozin‐treated rats, urinary glucose excretion was positively and significantly correlated with urine volume (Veh: *r* = .926, *p* < .001; Ipra: *r* = .629, *p* = .029) (Figure 5d) and there was a positive trend relationship between urinary glucose excretion and serum Na^+^ (Veh: *r* = −.701, NS; Ipra: *r* = .453, NS) (Figure 5e). In contrast, urinary glucose excretion in ipragliflozin‐treated rats was not associated with fluid balance (Veh: *r* = .454, NS; Ipra: *r* = .213, NS) (Figure 5f). In ipragliflozin‐treated diabetic rats, serum osmolality (*r* = .208, NS), serum glucose (*r* = .045, NS), serum Na^+^ (*r* = −.151, NS), serum Cl^−^ (*r* = .453, NS), serum K^+^ (*r* = −.282, NS), serum ([Na^+^] + [Cl^−^]) (*r* = .209, NS), and serum ([Na^+^] + [K^+^]) (*r* = −.202, NS) did not significantly correlate with urine vasopressin excretion.

### 3.3 Ipragliflozin treatment increased phosphorylation of AQP2 at Ser269 in diabetic GK rats

**Figure 6:**
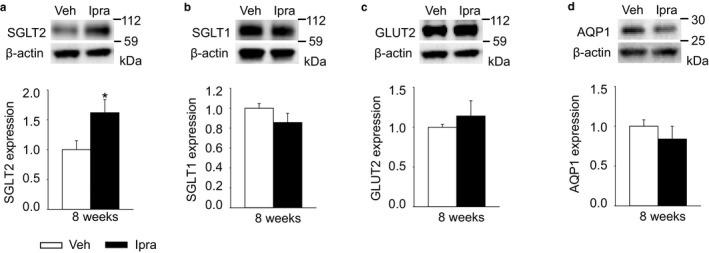
Renal membrane expression of transporters in diabetic GK rats with vehicle or ipragliflozin. (a and b) Ipragliflozin increased SGLT2 expression in diabetic GK rats, but did not change SGLT1 expression. (c and d) The expression of GLUT2 or AQP1 was not affected by ipragliflozin. Veh: normal diet (CE2, CLEA JAPAN Inc.); Ipra: normal diet containing ipragliflozin [100 mg/kg diet]. Values are expressed as means ± standard error; *n* = 4–10/group. *p < .05 versus Veh

**Figure 7:**
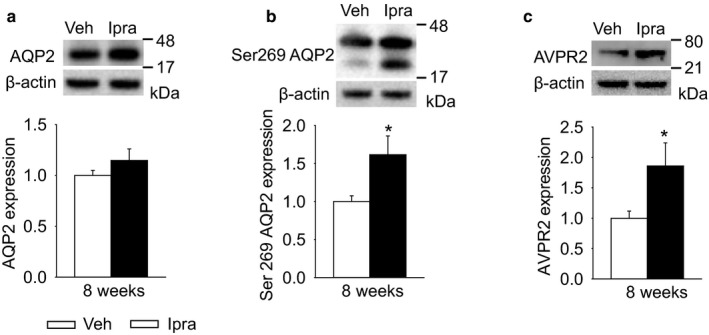
Renal membrane expression of transporters in diabetic GK rats treated with vehicle or ipragliflozin for 8 weeks. Ipragliflozin treatment numerically increased AQP2 expression (a), and significantly increased phosphorylation of AQP2 at Ser269 (b) and AVPR2 expression (c) in diabetic GK rats. Veh: normal diet (CE2, CLEA JAPAN Inc.); Ipra: normal diet containing ipragliflozin [100 mg/kg diet]. Values are expressed as means ± standard error; *n* = 4–10/group. *p < .05 versus Veh

Ipragliflozin increased the renal membrane SGLT2 expression in diabetic GK rats, without affecting the expression of SGLT1 (Figure 6a and b) or of the basolateral facilitative glucose transporter GLUT2 (Figure 6c). Upregulation of SGLT2 protein expression has previously been observed in response to SGLT2 inhibition (Masuda et al., 2018; Vallon et al., 2014). In contrast, upregulation of renal gluconeogenesis in mice lacking tubular NHE3 is associated with marked suppression of SGLT2 expression (Onishi et al., 2019), probably due to a negative feedback mechanism to prevent excessive intracellular glucose concentrations. In this regard, blocking SGLT2‐mediated glucose uptake would lower intracellular glucose levels and inhibit this negative feedback on SGLT2 expression. AQP1 is expressed and mediates fluid transport in the proximal tubule and the descending limb of Henle's loop (Vallon, Verkman, & Schnermann, 2000), and its expression was also similar between the two groups (Figure 6d). AQP2 is expressed in connecting tubules and cortical collecting ducts, where it regulates water excretion under the control of vasopressin (Xie et al., 2010). Total AQP2 protein expression was numerically increased with ipragliflozin treatment (Figure 7a). Ipragliflozin, however, increased the phosphorylation of AQP2 at Ser269 (Figure 7b), an indicator of vasopressin action and active AQP2 (Xie et al., 2010) and increased renal AVPR2 expression (Figure 7c).

### 3.4 Ipragliflozin treatment maintained body fluid volume in diabetic GK rats

**Figure 8:**
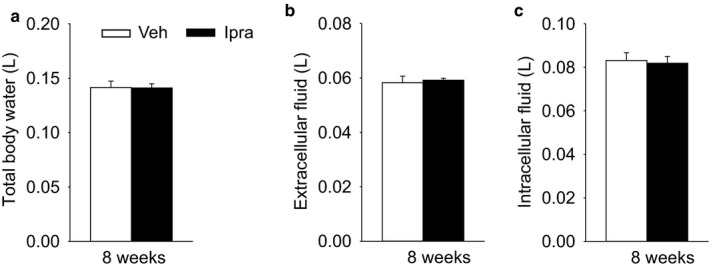
Ipragliflozin maintained total body water (a), extracellular fluid (b) and intracellular fluid (c) in diabetic GK rats. Veh: normal diet (CLEA Rodent Diet CE2); Ipra: normal diet containing ipragliflozin [100 mg/kg diet]. *p < .05 versus Veh. Values are expressed as means ± standard error; *n* = 4–5/group. *p < .05 versus Veh

Body fluid volume including total body water and extracellular and intracellular fluid determined by BIS were similar between the two groups (Figure 8a–c), consistent with the similar fluid balance (Figure 2c). These results suggest that SGLT2 inhibitor ipragliflozin maintained body fluid volumes in diabetic GK rats, confirming previous data obtained in another rat strain (Masuda et al., 2018).

## 4 DISCUSSION

This study showed that the SGLT2 inhibition with ipragliflozin induced a sustained glucosuria, diuresis, and natriuresis in moderately hyperglycemic GK rats. This was associated with increased vasopressin levels (as measured by urinary vasopressin excretion). The latter is expected to induce compensatory increases in fluid intake and renal water reabsorption, thereby preventing detectable changes in body fluid volume, as documented in this study. Vasopressin was negatively correlated with fluid balance, indicating that the osmotic diuresis induced by SGLT2 inhibition enhanced vasopressin tone and stimulated compensatory mechanisms of body fluid volume homeostasis.

The SGLT2 inhibitor ipragliflozin increased solute‐free water reabsorption with a positive correlation to urine vasopressin, which is expected to attenuate polyuria and body fluid depletion. Solute‐free water is the water without osmotic agents, and solute‐free water clearance is calculated by the formula: urine volume ‐ osmolar clearance. A negative value of solute‐free water clearance means that solute‐free water is reabsorbed in the tubule. In this study, ipragliflozin induced osmotic diuresis together with increased urinary glucose and Na^+^ excretion, and increased compensatory solute‐free water reabsorption. Without the latter compensatory mechanism, ipragliflozin might induce excessive urine flow rates that could accelerate body fluid depletion and result in renal dysfunction.

Vasopressin is a peptide hormone secreted from vasopressinergic nerve endings in the neurohypophysis, and plays an important role in regulating body fluid balance through fluid reabsorption in the renal collecting duct (Ettema et al., 2014; Ishikawa & Schrier, 2003; Knepper, Kwon, & Nielsen, 2015). In the current study, ipragliflozin increased urine vasopressin, which was positively associated with solute‐free water reabsorption. This poses the question, what triggered the release of vasopressin? The observed correlations suggest that the osmotic diuretic action, mainly due to glucosuria, the reduced fluid balance, and the lower blood pressure were main stimulators of vasopressin release during SGLT2 inhibition. In more detail, the osmotic diuretic action of ipragliflozin may have been a trigger of vasopressin release, because urinary glucose and Na^+^ excretion, which both contribute to the osmotic diuresis, were positively associated with urine vasopressin. The correlation was stronger for urinary glucose excretion than for urinary Na^+^ excretion. While the positive correlation between urinary Na^+^ concentration and vasopressin secretion was previously reported (Hew‐Butler, Noakes, Soldin, & Verbalis, 2010), the current study is the first to report a positive relationship between urinary glucose excretion and vasopressin excretion. These data suggest that the osmotic diuretic action mainly due to glucosuria stimulated vasopressin release. In our previous study in non‐diabetic Sprague–Dawley (*SD*) rats, ipragliflozin also increased urinary fluid, glucose and Na^+^ excretion in addition to urine vasopressin (Masuda et al., 2018). In contrast, ipragliflozin did not increase urine vasopressin in non‐obesity diabetic SDT rats, in which ipragliflozin did not change urinary fluid, glucose and Na^+^ excretion (Masuda et al., 2018). Second, the reduced fluid balance decreases circulating blood volume, which is a non‐osmotic stimulus for vasopressin secretion (Ettema et al., 2014; Ishikawa & Schrier, 2003). Third, the blood pressure reduction in ipragliflozin‐treated diabetic GK rats might play a role for the increase in vasopressin release, because hypotension is a potent mechanism of vasopressin release (Ettema et al., 2014; Ishikawa & Schrier, 2003). Blood pressure is a function of cardiac output and peripheral arterial resistance, and cardiac output is directly associated with the circulating volume of the venous return to the heart (Blaustein, Zhang, Chen, & Hamilton, 2006). Thus, a small reduction in circulating volume in ipragliflozin‐treated rats, potentially reflected by the small increase in hematocrit and serum Na^+^ and Cl^−^, might contribute to blood pressure reduction.

In general, elevation of circulating osmolality is a main trigger for releasing vasopressin (Ettema et al., 2014; Ishikawa & Schrier, 2003), but serum osmolality in this study was not different between the diabetic rats with and without ipragliflozin. However, among the determinants of serum osmolality, serum Na^+^ and Cl^−^ was higher and serum glucose was lower in the diabetic GK rats treated with ipragliflozin, suggesting that the similar total values of serum osmolality were an offset result. Previous studies demonstrated that vasopressin release is elevated in hyperglycemic patients and experimental diabetic animals (Iwasaki, Kondo, Murase, Hasegawa, & Oiso, 1996; Vokes, Aycinena, & Robertson, 1987; Zerbe, Vinicor, & Robertson, 1979). In the insulin‐deficient state of type 1 diabetes, blood glucose acts as an important stimulus for vasopressin release, because glucose transport into the osmosensor cells is dependent on insulin, and thus hyperglycemia induces an osmotic gradient between outside and inside of the osmoreceptor cells (Vokes et al., 1987). In contrast, glucose has been proposed not to be an effect osmolyte in normoglycemia and in hyperglycemia with intact insulin (Iwasaki et al., 1996; Sladek & Knigge, 1977; Thrasher, Brown, Keil, & Ramsay, 1980; Zerbe & Robertson, 1983), which should apply to type 2 diabetic GK rats. In the latter, ipragliflozin induced higher urine vasopressin associated with lower serum glucose levels, indicating that a change in serum glucose levels was not the stimulator of vasopressin release during SGLT2 inhibition, leaving the small increases in serum Na^+^ and Cl^−^ as alternative mechanisms. Thus, the most convincing mechanism to explain the correlation between urinary glucose excretion and vasopressin secretion is that the osmotic diuretic effect of glucose enhances water loss and causes a small increase in serum Na^+^ concentration, which then triggers vasopressin release. This mechanism was suggested by the positive relationship between urinary glucose excretion and serum Na^+^ levels.

The higher vasopressin levels in ipragliflozin‐treated GK rats were associated with increased phosphorylation of AQP2 at Ser269. AQP2 is expressed throughout the collecting‐duct system where vasopressin regulates osmotic transport of water (Knepper et al., 2015). Regulation of AQP2 by vasopressin is a result of vasopressin V2 receptor activation which triggers cyclic AMP‐dependent activation of a protein kinase network that causes increased transcription of AQP2, changes in its phosphorylation status, and redistribution of AQP2 to the luminal membrane (Knepper et al., 2015). The vasopressin‐induced increases in AQP2 phosphorylation at Ser269 has been proposed to be a more effective indicator of vasopressin activity than phosphorylation at Ser256 (Ando et al., 2018; Xie et al., 2010). In the current study, ipragliflozin increased vasopressin levels, the renal expression of the V2 vasopressn receptor, the phosphorylation of AQP2 at Ser269, and solute‐free water reabsorption, consistent with the notion that iplagliflozin‐stimulated vasopressin release increased free water reabsorption, at least in part, by AQP2 phosphorylation at Ser269.

SGLT2 inhibitor ipragliflozin increased urine volume, but maintained body fluid volume in GK rats, as we recently reported (Masuda et al., 2018). A recent human study of non‐hypervolemic type 2 diabetic patients showed that SGLT2 inhibitors empagliflozin or dapagliflozin maintained extracellular fluid volume for 6 months with a transient short‐term fluid reduction (Schork et al., 2019). Similarly, another study in type 2 diabetic patients reported that empagliflozin induced a transient short‐term diuresis and negative fluid balance but did not change long‐term overall fluid balance (Yasui et al., 2018). Thus, while the body fluid maintenance by SGLT2 inhibitors has recently been demonstrated in human studies, the current study provided first evidence for compensatory mechanisms that include vasopressin‐induced free water reabsorption in addition to increased fluid intake. The current data were obtained at 8 weeks of ipragliflozin treatment, when the animals are expected to be in steady state with regard to drug concentrations and effects and compensatory body responses. Therefore, we would expect the observed effects to last with longer treatment, unless the basic physiology changes (e.g., due to development of kidney disease).

The homeostatic mechanisms to maintain body fluid volume are likely important for the renal safety of SGLT2 inhibitors. Recent large‐scale clinical trials and meta‐analysis have shown that SGLT2 inhibitors may even protect type 2 diabetic patients from AKI, although an increase in AKI related to the diuretic property and resulting volume depletion have been a concern (Donnan et al., 2019; Gilbert & Thorpe, 2019). In contrast, the commonly used and more potent loop diuretics decrease body fluid volume, as shown in euvolemic rats (Hu et al., 2012), and may worsen renal function due to body fluid reduction in chronic kidney disease patients (Khan, Sarriff, Adnan, Khan, & Mallhi, 2017). SGLT2 inhibitors exhibit an initial transient diuretic peak within the first week followed by only a moderate sustained diuresis (Masuda et al., 2017; Tanaka et al., 2017; Yasui et al., 2018). The present results indicate an important role for vasopressin in this regard as it attenuates the diuretic action of SGLT2 inhibitors and maintains body fluid volume by stimulating fluid intake.

In conclusion, the SGLT2 inhibitor ipragliflozin induced a sustained osmotic diuresis with compensatory increases in vasopressin‐induced solute‐free water reabsorption via phosphorylation of AQP2 at Ser269 to maintain body fluid volume. Furthermore, vasopressin was inversely correlated with fluid balance, indicating that the osmotic diuresis induced by SGLT2 inhibition stimulates compensatory fluid reabsorption as a homeostatic mechanism to maintain body fluid volume.

## CONFLICT OF INTEREST

Astellas Pharma (Tokyo, Japan) provided SGLT2 inhibitor ipragliflozin for this study. Over the past 36 months, V.V. has served as a consultant and received honoraria from Bayer, Boehringer Ingelheim, Astra‐Zeneca, Intarcia Therapeutics, Janssen Pharmaceutical, Eli Lilly, and Merck, and received grant support for investigator‐initiated research from Boehringer Ingelheim, Astra‐Zeneca, Bayer, Fresenius, and Janssen.

## AUTHOR CONTRIBUTIONS

T.M., S.M., and V.V. conceptualized and designed the project; T.M., K.F., M.W., and Y.W., performed animal experiments; T.M., K.F., and M.W. analyzed the data; T.M., S.M., K.F., M.W. Y.W., K.O., V.V., and D.N. interpreted the results; T.M. prepared figures for the manuscript; H.K. provided antibodies (SGLT2 and SGLT1 antibodies); T.M. and V.V. drafted the manuscript; T.M., S.M., K.F., M.W., Y.W., K.O., H.K., V.V., and D.N. edited and revised the manuscript; T.M., S.M., K.F., M.W., Y.W., K.O., H.K., V.V., and D.N. approved the final version of manuscript.
