# Supplementary figures and images for: Osmotic diuresis by SGLT2 inhibition stimulates vasopressin‐induced water reabsorption to maintain body fluid volume
Source: Physiol Rep. 2020 Jan 28;8(2):e14360. doi: 10.14814/phy2.14360 (PMC6987478; doi:10.14814/phy2.14360)

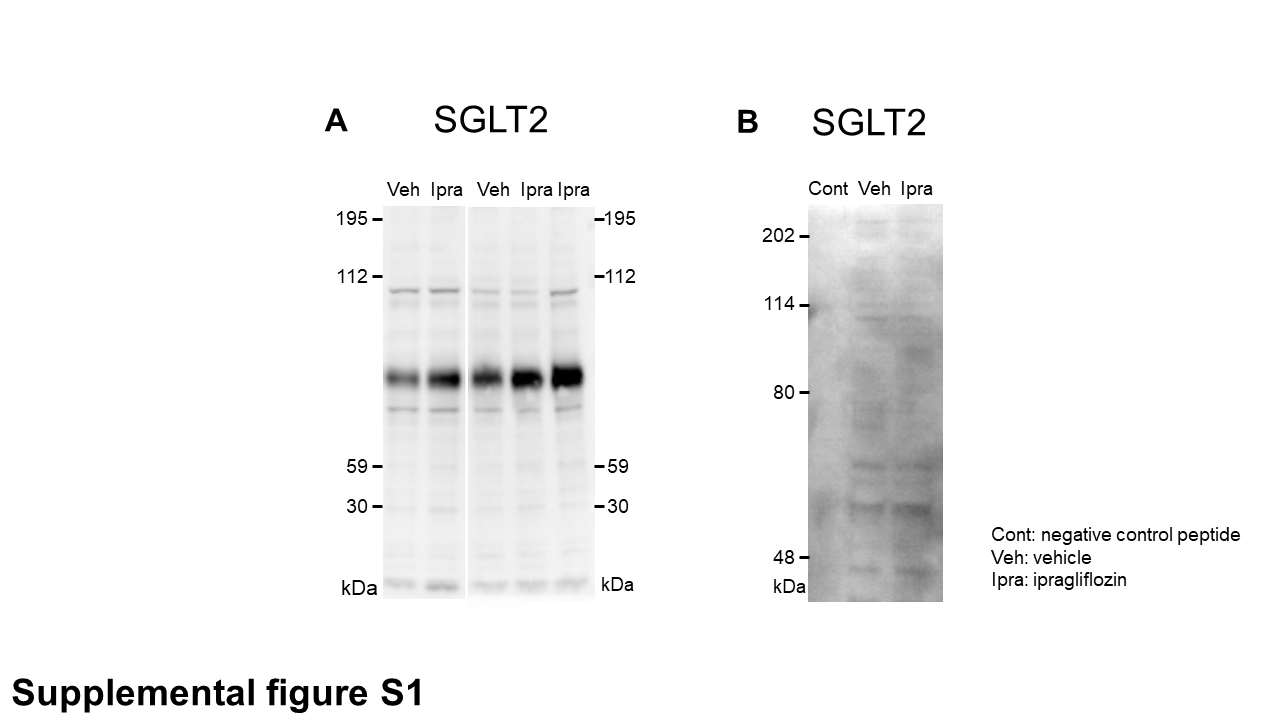

Supplement: Supplementary file 1 [file PHY2-8-e14360-s001.tif]

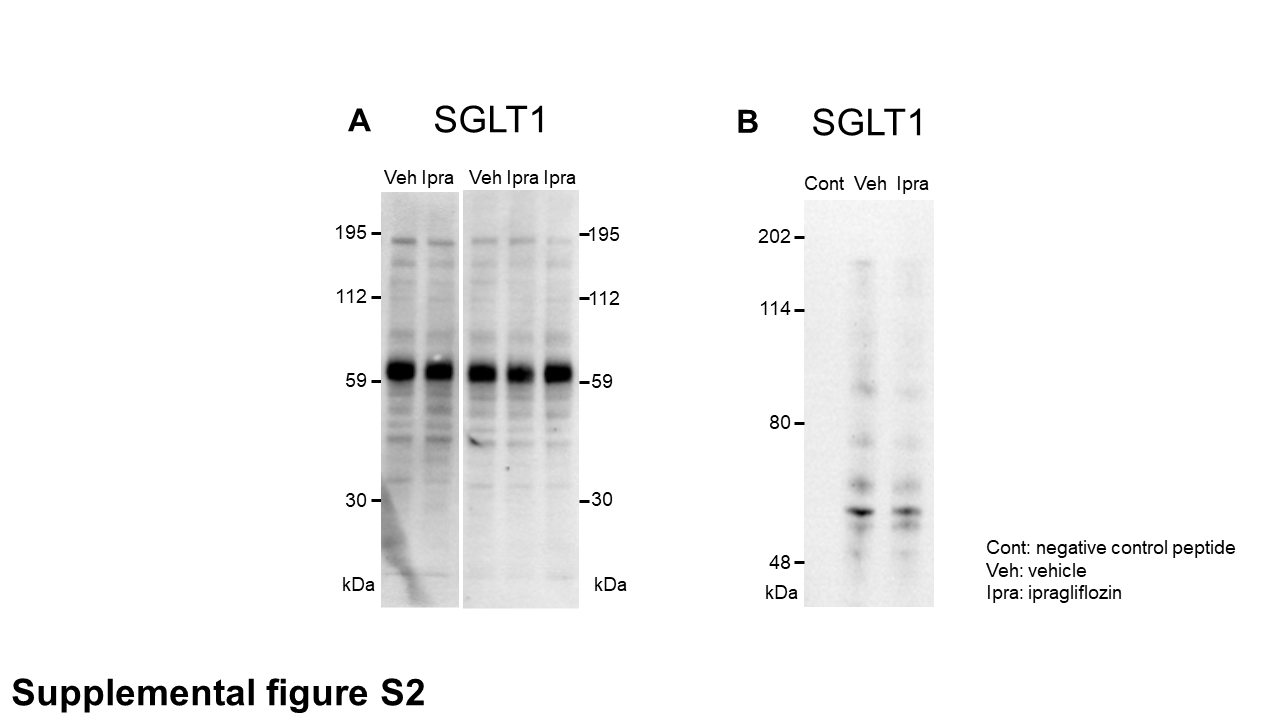

Supplement: Supplementary file 2 [file PHY2-8-e14360-s002.tif]
